# Lymphoepithelial cyst in the palatine tonsil: A case report and literature review

**DOI:** 10.1097/MD.0000000000029246

**Published:** 2022-05-27

**Authors:** Jae Yeon Moon, Namkyun Kim, Ji Yun Jeong, Jung Soo Kim, Sung Jae Heo

**Affiliations:** a Department of Otorhinolaryngology-Head and Neck Surgery, School of Medicine, Kyungpook National University, Daegu, South Korea; b Internal Medicine, School of Medicine, Kyungpook National University, Daegu, South Korea; c Pathology, School of Medicine, Kyungpook National University, Daegu, South Korea.

**Keywords:** cysts, epithelium, lymphoid tissue, palatine tonsil

## Abstract

**Rationale:**

Lymphoepithelial cyst of the oral cavity is very rare. Most intraoral lymphoepithelial cysts are observed in the floor of the mouth and very few cases have been reported of its occurrence in the palatine tonsil.

**Patient concerns:**

A 37-year-old healthy woman with no remarkable medical history visited our department with a complaint of frequent tonsillitis.

**Interventions:**

On endoscopic examination, yellowish mass was observed in the palatine tonsil and removed via an intraoral approach. The mass was completely removed with the left palatine tonsil.

**Diagnoses & Outcomes:**

Histopathological examination and immunohistochemical staining confirmed a Lymphoepithelial cyst.

**Lessons:**

Lymphepithelial cysts of the palatine tonsils are extremely rare and are easily overlooked because there are few reported cases. Therefore, care must be taken when examining the tonsil mass.

## 1 Introduction

Lymphoepithelial cyst is a relatively rare lesion that develops within lymphoid tissue and occurs in various sites. An intraoral lymphoepithelial cyst usually presents as an asymptomatic mass and is detected on routine physical examination. It is easily misdiagnosed as lipoma, fibroma, dermoid cyst, and so on. Its histological findings are characterized by a cystic lesion lined with stratified squamous epithelium surrounded by lymphoid tissues. Surgical resection is recommended if it is symptomatic, and lymphoepithelial cyst has minimal risk of recurrence and malignant change.

According to Xi Yang et al,^[1]^ the most common sites for an intraoral lymphoepithelial cyst are the tongue and floor of the mouth, which account for 88.3% of cases. Lymphoepithelial cyst in the palatine tonsil is extremely rare and very few cases have been reported. This article presents a case of lymphoepithelial cyst in the palatine tonsil with a review of previously reported cases. This study was approved by the institutional review board of Kyungpook National University Chilgok Hospital.

## 2 Clinical report

**Figure 1:**
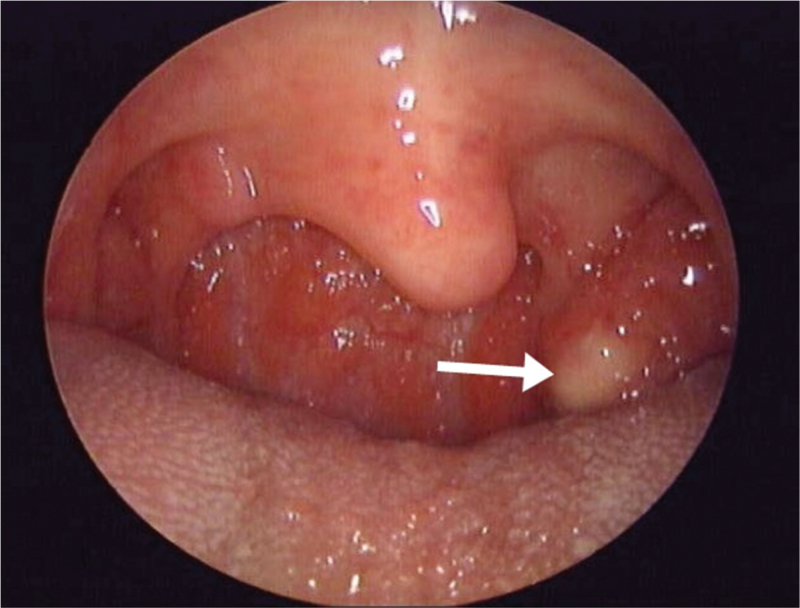
On the laryngoscopic examination, a yellowish mass lesion was observed in upper portion of the left palatine tonsil.

A 37-year-old healthy woman with no remarkable medical history visited our department with a complaint of frequent tonsillitis. On physical examination, the left palatine tonsil was Friedman grade III and a yellowish lesion protruded in the medial portion of the palatine tonsil (Fig. 1). There was no pain and tenderness in the portion of left palatine tonsil.

**Figure 2:**
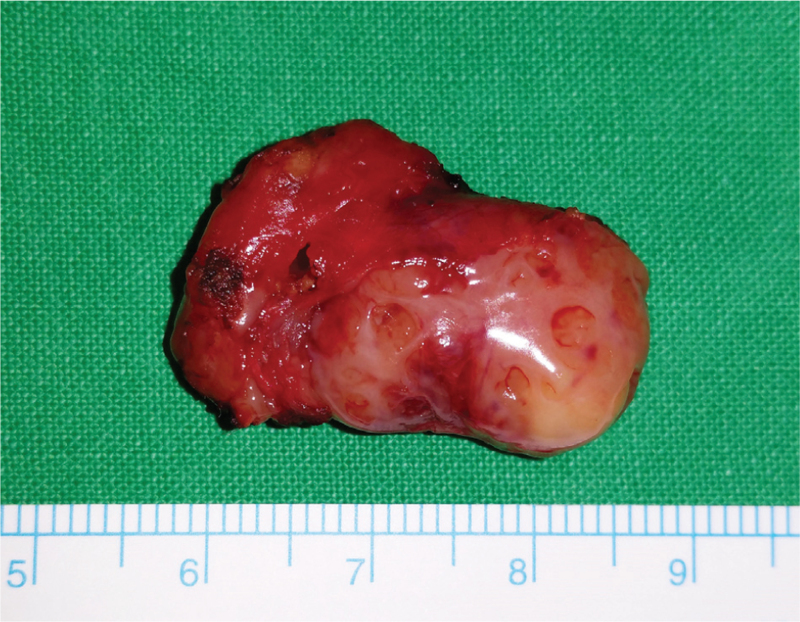
The mass was oval and well encapsulated, 2.5 × 1.5 cm in size.

**Figure 3:**
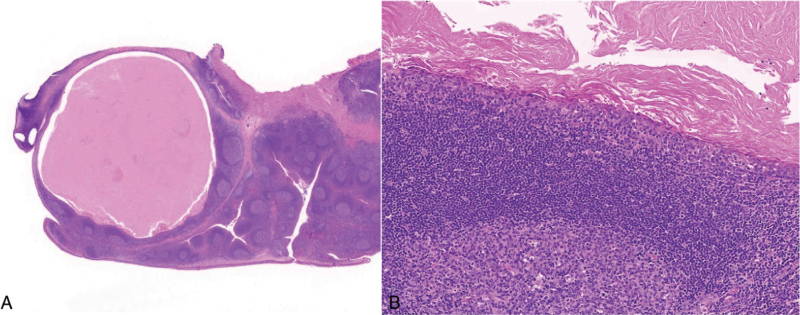
(A) Intratonsillar cystic mass 0.7 cm in size (×5, H&E stain), (B) The keratin material that filled the cyst was lined with stratified squamous epithelium and surrounded by lymphoid tissue with follicular lymphoid hyperplasia (×100, H&E stain).

The patient underwent tonsillectomy on both sides to treat frequent tonsillitis and diagnose the yellowish lesion. The mass was completely removed with the left palatine tonsil; the size of mass was 2.5 × 1.5 cm (Fig. 2). Upon histopathological examination, the cystic mass was found to have keratin material inside (Fig. 3). The cystic wall was lined with stratified squamous epithelium and some lymphocytes infiltration was noted in the epithelium, which showed intraepithelial lymphocytosis. The cyst was surrounded by lymphoid tissue with follicular lymphoid hyperplasia. These findings were consistent with lymphoepithelial cyst. There were no remarkable side effects after surgery and no recurrence during the 40 months’ follow-up period.

## 3 Discussion

Lymphoepithelial cysts account for only 0.09% of all oral biopsy specimens.^[2,3]^ Since palatine tonsil involvement is extremely rare and clinical characteristics are non-specific, many clinicians misdiagnose intraoral lymphoepithelial cyst as a mucocele, dermoid cyst, lipoma, and so on.^[3]^ Therefore, pathological confirmation is crucial for diagnosing lymphoepithelial cyst in the oral cavity. There are several theories on the pathogenesis of lymphoepithelial cysts, but Knapp's theory is the most accepted.^[3]^ He proposed that pseudocysts develop when the tonsillar crypt opening becomes plugged, which results in enlargement of the tonsillar tissue secondary to an accumulation of purulent materials or desquamated cells and keratin.

To our knowledge, 6 cases of intraoral lymphoepithelial cyst in the palatine tonsil have been reported in the literature (Table 1). The age of patients, 4 males and 2 females, ranged from 10 to 72 years old. Most patients complained of swallowing difficulty and lymphoepithelial cysts were mostly found in the right side of the palatine tonsil. The size of mass was 1.5 to 2 cm in diameter.^[4–6]^

**Table 1:** Clinical characteristics of lymphoepithelial cysts in the palatine tonsils.

Author	Report year	Gender	Age	Location	Chief complaint	Size (cm)
Bingol et al^[4]^	2016	Female	66	Rt tonsil	Dysphagia	NA
Castro et al^[5]^	2015	Male	21	Rt tonsil	Painless lump	1.5 cm
Choi et al^[6]^	2010	Female	30	Lt tonsil	Globus sensation	1.5 cm
Mahdavi et al^[7]^	2013	Male	72	Both tonsils	Snoring	NA
Kwon et al^[8]^	2006	Male	10	Rt tonsil	Snoring	NA
Tanaka et al^[9]^	2004	Male	55	Rt tonsil	Dysphagia	2 cm

Lt = left, NA = not available, Rt = right.

The age of the patients with lymphoepithelial cyst in the palatine tonsil was within the range of that of patients with intraoral lymphoepithelial cysts. However, unlike Xi Yang's analysis of intraoral lymphoepithelial cyst cases,^[1]^ male patients with lymphoepithelial cyst in the palatine tonsil outnumbered females by 2 to 1. Moreover, there were differences in the size of the lesions and patients’ chief complaints. The size of the lesions in the palatine tonsil was mostly greater than 1.5 cm in diameter, while most intraoral lymphoepithelial cysts are smaller than 1 cm in diameter. In addition, patients with lymphoepithelial cyst in the palatine tonsil were symptomatic, mainly complaining of difficulty in swallowing. However, most patients with intraoral lymphoepithelial cysts were asymptomatic. Therefore, differences in size and location of lymphoepithelial cysts may be responsible for symptoms in the patients.

Compared to other cases of lymphoepithelial cyst in the palatine tonsil, our case is notable for the size of the mass, which was about 2.5 cm in diameter. However, the chief complaint of frequent tonsillitis was not related the effect of the mass. There are no reports of neoplastic transformation or recurrence after surgical excision of these cysts.^[3]^

## Author contributions

**Conceptualization:** Sung Jae Heo.

**Data curation:** Jae Moon, Ji Jung, Jung Kim, Namkyun Kim, Sung Jae Heo.

**Formal analysis:** Jae Moon, Ji Jung, Jung Kim, Namkyun Kim, Sung Jae Heo.

**Investigation:** Jae Moon, Ji Jung, Jung Kim, Namkyun Kim, Sung Jae Heo.

**Methodology:** Jae Moon, Ji Jung, Namkyun Kim, Sung Jae Heo.

**Supervision:** Jung Kim, Sung Jae Heo.

**Validation:** Jung Kim, Sung Jae Heo.

**Writing – original draft:** Jae Moon, Namkyun Kim.

**Writing – review & editing:** Sung Jae Heo.
